# Prediction of Mini-Mental State Examination Scores for Cognitive Impairment and Machine Learning Analysis of Oral Health and Demographic Data Among Individuals Older Than 60 Years: Cross-Sectional Study

**DOI:** 10.2196/75069

**Published:** 2025-08-25

**Authors:** Alper Idrisoglu, Johan Flyborg, Sarah Nauman Ghazi, Elina Mikaelsson Midlöv, Helén Dellkvist, Anna Axén, Ana Luiza Dallora

**Affiliations:** 1 Department of Health, Blekinge Institute of Technology, Valhallavägen 1, Karlskrona, 371 41, Sweden, 46 701462619

**Keywords:** classification, machine learning, mini-mental state examination, cognitive impairment, oral health

## Abstract

**Background:**

As the older population grows, so does the prevalence of cognitive impairment, emphasizing the importance of early diagnosis. The Mini-Mental State Examination (MMSE) is vital in identifying cognitive impairment. It is known that degraded oral health correlates with MMSE scores ≤26.

**Objective:**

This study aims to explore the potential of using machine learning (ML) technologies using oral health and demographic examination data to predict the probability of having MMSE scores of 30 or ≤26 in Swedish individuals older than 60 years.

**Methods:**

The study had a cross-sectional design. Baseline data from 2 longitudinal oral health and ongoing general health studies involving individuals older than 60 years were entered into ML models, including random forest, support vector machine, and CatBoost (CB) to classify MMSE scores as either 30 or ≤26, distinguishing between MMSE of 30 and MMSE ≤26 groups. Nested cross-validation (nCV) was used to mitigate overfitting. The best performance-giving model was further investigated for feature importance using Shapley additive explanation summary plots to easily visualize the contribution of each feature to the prediction output. The sample consisted of 693 individuals (350 females and 343 males).

**Results:**

All CB, random forest, and support vector machine models achieved high classification accuracies. However, CB exhibited superior performance with an average accuracy of 80.6% on the model using 3 × 3 nCV and surpassed the performance of other models. The Shapley additive explanation summary plot illustrates the impact of factors on the model’s predictions, such as age, Plaque Index, probing pocket depth, a feeling of dry mouth, level of education, and use of dental hygiene tools for approximal cleaning.

**Conclusions:**

The oral health parameters and demographic data used as inputs for ML classifiers contain sufficient information to differentiate between MMSE scores ≤26 and 30. This study suggests oral health parameters and ML techniques could offer a potential tool for screening MMSE scores for individuals aged 60 years and older.

## Introduction

The anticipated global population of 8 billion individuals for 2023 has been achieved. The population growth continues to persist, albeit experiencing a decelerated trajectory. Global birth rates are declining, juxtaposed with a consistent upward trajectory in average life expectancy, attributed to advancements in scientific knowledge, improved nutritional standards, advancements in public health infrastructure, and enhanced sanitation practices [1]. An estimate for the year 2050 suggests that individuals aged 60 years and older will constitute 38% of the total population and will continue to increase. While many individuals enjoy several quality years of life, a significant portion of them will also struggle with neurocognitive diseases such as Alzheimer disease (AD) and mild cognitive impairment (MCI) [2]. Until now, diagnosing AD, which is the most common neurocognitive disorder, has required extensive hospital-level efforts, and no curative treatment has been available [3]. Recent research has identified biomarkers in blood that identify proteins associated with AD [4]. At the same time, several approved antibody medications now stop the breakdown of nerve cells caused by phosphorylated Tau and amyloid plaques [5]. This is expected to significantly burden the health care system, as early diagnosis is essential for a good treatment result. The need for screening methods is expected to be extensive. It is widely recognized that MCI and neurocognitive diseases contribute to deteriorating oral health, with individuals affected by these conditions exhibiting poorer oral health [6,7]. The decline in oral health carries profound implications, causing hardship for affected individuals and placing substantial demands on society and health care systems in terms of both costs and human resources. However, recent research suggests that the deterioration of oral health in individuals with MCI can be delayed by at least 12 months through the introduction of a powered toothbrush [8]. Additionally, severe periodontitis poses a risk factor for dementia [9]. Various instruments are available to assess cognitive function, with one of the most commonly used being the Mini-Mental State Examination (MMSE) test, which is internationally recognized and validated in numerous languages. The MMSE scale ranges from 0 to 30, with higher scores indicating a better cognitive function [10]. Recently, machine learning (ML) has emerged as a valuable aid in enhancing the precision and efficiency of cognitive assessments [11,12], leading to significant progress in the field of cognitive research [13-15]. Various datasets and algorithms can be used to better predict MMSE scores, differentiate between MCI and AD, and improve cognitive test batteries. A comprehensive study used a multifaceted approach, integrating brain cortical characteristics, biological markers, risk factors, positron emission tomography scan measures, and cognitive scores into a joint feature set to predict MMSE scores at 6 and 12 months, achieving mean absolute errors of 1.40 [16]. Researchers have improved predictive accuracy by experimenting with ML algorithms, including logistic regression, decision trees, support vector machine (SVM), XGBoost, and random forests (RF). This approach enhances diagnostic precision and offers valuable insights into disease progression and treatment response [17]. Similarly, previous research has explored integrating MRI data with cognitive tests, such as logical memory, to detect and distinguish individuals with normal cognition from those with MCI. Through deep learning models trained on MRI slices and fusion techniques, the combined model surpassed individual modalities in predictive performance, underscoring the potential of multimodal fusion in cognitive assessment, achieving an accuracy of 90.9% [18]. Earlier research has explored the area of oral health and its relationship with cognitive health. One meta-analysis [19] found that oral health in individuals with dementia was significantly worse than that of the controls. Another systematic literature review [20] came to a similar conclusion, attributing this to both difficulties in self-care due to the consequences of the disease and inflammatory mechanisms. Further, a systematic literature review [21] also indicated an association between oral health and cognitive impairment, implying a bidirectional relationship between these and ratifying a need for higher-level evidence in this area. Another systematic literature review analyzed the effects of oral interventions on cognition and found that dental treatments had a subjective influence on cognition [22]. One study [23] explored the relationship between oral health–related quality of life and cognitive function among individuals in residential care settings. Significant correlations were found between poor oral health and cognitive impairment, including MCI, revealing a relationship between cognitive status and perceived oral health [23]. Another study investigated the use of noninvasive digital biomarkers to quantify oral health and applied ML algorithms to detect cognitive decline within a community setting. Significant findings included notable differences in oral diadochokinesis rates and oral acidity between individuals with cognitive decline and those with normal cognitive function. This further demonstrates that digital oral health biomarkers have the potential to track cognitive function and facilitate early detection of cognitive decline [24].

Despite promising advancements in clinical practice and the possibility of early diagnosis, further research is needed. One significant research gap is the lack of exploration of oral health parameters as potential indicators for estimating MMSE scores. Although oral health has a recognized influence on overall well-being and cognitive function, there have been no empirical studies to explore the integration of oral health metrics into ML-based cognitive assessment models. This opens an opportunity for future studies to expand the scope of cognitive assessments and develop more comprehensive diagnostic frameworks. Many individuals routinely visit dental care facilities for treatment and preventive care. Leveraging existing patient history data alongside current examination findings requires minimal additional effort from dental care staff, and it can be seamlessly integrated with digital medical record systems. Previous research presents diverse evidence on the association between oral and cognitive health. This study builds on this knowledge by investigating different parameters and using a robust and balanced sample of participants in the MMSE 30 and MMSE ≤26 score groups.

Integrating oral health parameters into ML-driven decision support systems for MMSE score estimation could offer insights into the interplay between oral health and cognitive function. By leveraging advanced algorithms and comprehensive datasets encompassing cognitive and oral health data, researchers could uncover novel biomarkers and risk factors for cognitive decline, paving the way for more personalized approaches to cognitive assessment and intervention.

This study aims to explore the potential of using ML technologies using oral health and demographic examination data to predict the probability of having MMSE scores of 30 or ≤26 in Swedish individuals older than 60 years.

The main contributions of this study are as follows:

- The evaluation of the potential of oral health parameters for binary classification of MMSE scores of 30 and ≤26.
- The evaluation of the most deterministic oral health parameters that influence the outcome of the best-performing ML classifier.
- The assessment of RF, SVM, and CatBoost (CB) ML classifiers as indicators of MMSE scores of 30 and ≤26.

## Methods

### Ethical Considerations

This study used secondary, open-access data from the Swedish National Study on Aging and Care (SNAC-B) and Support Monitoring and Reminder Technology for Mild Dementia (SMART4MD) projects. No new data were collected specifically for this study. The use of SNAC-B data was approved by the Ethics Committee of Lund University (LU 604‐00). The data for SMART4MD was collected and used under the approval of the Ethical Review Board in Sweden (LU No. 650‐00 and No. 744‐00). Informed consent was obtained from all participants in both studies. All data were fully anonymized prior to access by the authors. No compensation was provided to participants for the purpose of this study. All data were used in accordance with applicable data sharing agreements and stored securely. The study complies with the ethical standards outlined in the Declaration of Helsinki.

### Data Description

The formed sample was used in experiments with ML models, including RF, SVM, and CB, aiming to indicate whether the participant’s MMSE scores were 30 or ≤26. Data from 2 studies, which recruited participants from the European collaborative study SMART4MD [25] and the SNAC-B [26], formed the sample. SNAC-B is a longitudinal study involving individuals aged 60 years or older. Participants undergo examinations every 6 years, with more frequent assessments every 3 years, starting at the age of 78 years. The cohort ranges from 60 to 96 years of age [26]. The study sample recruited from SMART4MD involved individuals aged 60 years or older, examined every 6 to 12 months over 36 months [27]. Both studies are conducted at a research clinic affiliated with Blekinge Institute of Technology by experienced dentists and dental hygienists and involve comprehensive clinical and demographic data collection. Ethical approval was obtained for both studies, and informed consent was collected from all participants. While both studies are longitudinal in design, only the baseline visit data, meaning the first available observation per participant, were used in this study.

All clinical and demographic features, including oral health variables, were extracted from each participant’s first examination. This resulted in a cross-sectional dataset suitable for ML analysis. The final sample included 693 baseline observations (350/693, 51% females and 343/693, 49% males) with an average age of 75 years. The sample was divided into 2 relatively balanced groups: one with MMSE scores of ≤26 and one with an MMSE score of 30. Observations with intermediate scores of 27‐29 were excluded to ensure distinct group separation. There was a total of 339/693 (49%) observations for individuals with ≤26 MMSE scores (165/339, 49% females and 174/339, 51% males) and 354/693 (51%) observations for the MMSE 30 score (185/354, 52% females and 169/354, 48% males). The dataset comprised 693 observations, each with 16 features (Table 1). The oral health features selected for inclusion were limited to those available in both datasets and supported by prior studies linking them to general health or cognitive decline. Examples include the Plaque Index (PI), which reflects hygiene maintenance; dry mouth, often associated with medication use and systemic conditions; and the number of teeth, which may serve as a proxy for long-term oral care and functional ability.

The listed features represent a combination of demographic, behavioral, and clinical oral health indicators obtained from baseline dental examinations. Probing pocket depth (PPD) measurements were categorized based on severity: values of 4 mm typically reflect a mild periodontal status, 5 mm indicate moderate depth, and ≥6 mm signify advanced periodontal disease. Variables such as PPD, PI, and bleeding on probing (BOP) were calculated as the percentage of affected surfaces out of the total examined. Self-reported variables, including dry mouth and hygiene tool use, were assessed through standardized questionnaires. Denture status was recorded separately for full and partial prostheses in both the maxillary and mandibular arches. These variables were selected due to their availability in both datasets and their clinical relevance to cognitive and systemic health in older adults.

MMSE scores are known to vary within individuals over time. Research indicates that changes of up to 3 points may occur without representing a clinically meaningful cognitive change, especially in older populations [28]. To reduce misclassification due to such variability, only individuals with MMSE scores of ≤26 or exactly 30 were included in the dataset. Based on this analogy, the MMSE ≤26 and the MMSE 30 score groups were designated and labeled as “1” and “0,” respectively. No normalization, mean-centering, or outlier filtering was performed prior to model training. Tree-based models like RF and CB are scale-invariant, and for consistency, unscaled features were also used with SVM.

**Table 1.** List of features used for the study.

Parameter	Description	Index/Categories
Age	Participant age	Number
Education	Level of education	Elementary School Secondary School Higher education
PI^a^	Number of tooth surfaces in % with dental plaque of the total number of surfaces	Percentage
PPD^b^ 4mm	The number of PPD 4 mm in % of the total number of surfaces	Percentage
Use of dental hygiene tools for approximal cleaning	How often do you use approximal dental hygiene tools?	Rarely/never Once a week Daily Several times daily
Gender	Gender	Male, female
A feeling of dry mouth	Do you experience a sensation of dry mouth?	Yes, often Yes sometimes No never
Number of teeth	Number of teeth	Number
PPD 5mm	The number of PPD 5 mm in % of the total number of surfaces	Percentage
Mirror test	Mirror test	Glides easily Sliding Getting stuck
BOP^c^	The number of surfaces with BOP in % of the total number of surfaces.	Percentage
PPD≥6mm	The number of PPD ≥ 6 mm in % of the total number of surfaces	Percentage
Mandibular denture	Mandibular denture	Yes, No
Maxillary denture	Maxillary denture	Yes, No
Mandibular partial denture	Mandibular partial denture	Yes, No
Maxillary partial denture	Maxillary partial denture	Yes, No

a PI: Plaque Index.

b PPD: probing pocket depth.

c BOP: bleeding on probing.

### Classification Experiment

Several well-known classifiers, RF, CB, and SVM, were used for the classification experiment. The dataset was split into 2 subgroups: a training set comprising 80% of the data (554/693 samples) and a test set containing 20% (139/693 samples). To ensure an unbiased estimation of model performance and to reduce the risk of overfitting during hyperparameter tuning, nested cross-validation (nCV) was applied within the training data.

The parameter grid for hyperparameter tuning was defined as follows:

- For Random Forest:{'n_estimators’: [50, 100, 200],‘max_depth’: [None, 10, 20], ‘min_samples_split’: [2,5,10]}
- For SVM: {'C’: [0.1, 1, 10],'kernel’: ['linear’, ‘rbf,’ ‘poly’], ‘degree’: [2, 3, 4]}
- For CatBoost:{‘iterations’: [100, 200, 300], ‘learning_rate’: [0.01, 0.1, 0.2], ‘depth’: [4, 6, 8], ‘l2_leaf_reg’: [1, 3, 5]}

nCV is particularly appropriate in ML pipelines that involve hyperparameter optimization or model selection. It separates the parameter tuning step from the performance evaluation step, which prevents information leakage between training and test data and avoids optimistically biased performance estimates. This methodological rigor is especially important in moderate-sized datasets where variance due to overfitting can significantly affect the results [29-31].

Model performance was assessed using a 10 × 10 nCV framework. The outer 10-fold loop was used to evaluate generalization performance, while the inner loop was used solely for hyperparameter optimization. This design avoids information leakage and produces unbiased performance estimates, allowing for a robust assessment of model performance under varying fold configurations and providing a stable basis for comparison across classifiers.

### Performance Evaluation

To evaluate the performance of each ML classifier, multiple metrics were used, including accuracy, precision, recall, and *F*_1_-score. To enhance clinical interpretability, the following definitions were used: true positives (TP) represent correctly identified positive cases; false positives (FPs) refer to negative cases incorrectly classified as positive; true negatives (TN) are correctly identified negative cases; and false negatives (FN) denote positive cases that were incorrectly classified as negative. Box plots, which illustrate the distribution of accuracy scores for the respective models and additional statistical information, were used for the data visualization. Subsequently, the classifier with the highest scores was further analyzed for its feature importance. The examination of the most essential features relied on Shapley Additive Explanation (SHAP) summary plots, providing visualizations of the influence of parameter groups on the output, organized by their importance. This importance is based on SHAP values, offering insights into features’ effects on the ML model’s decision-making process [32].

### Experimental Setup

The experiment was conducted on a Dell Precision 7920 MT desktop, and all models were implemented using Python (version 3.9; Python Software Foundation) with Scikit-learn (version 1.2.2) and CB (version 1.2). Visualization was supported via Seaborn (version 0.12.2).

## Results

### Experimental Results

**Figure 1.**
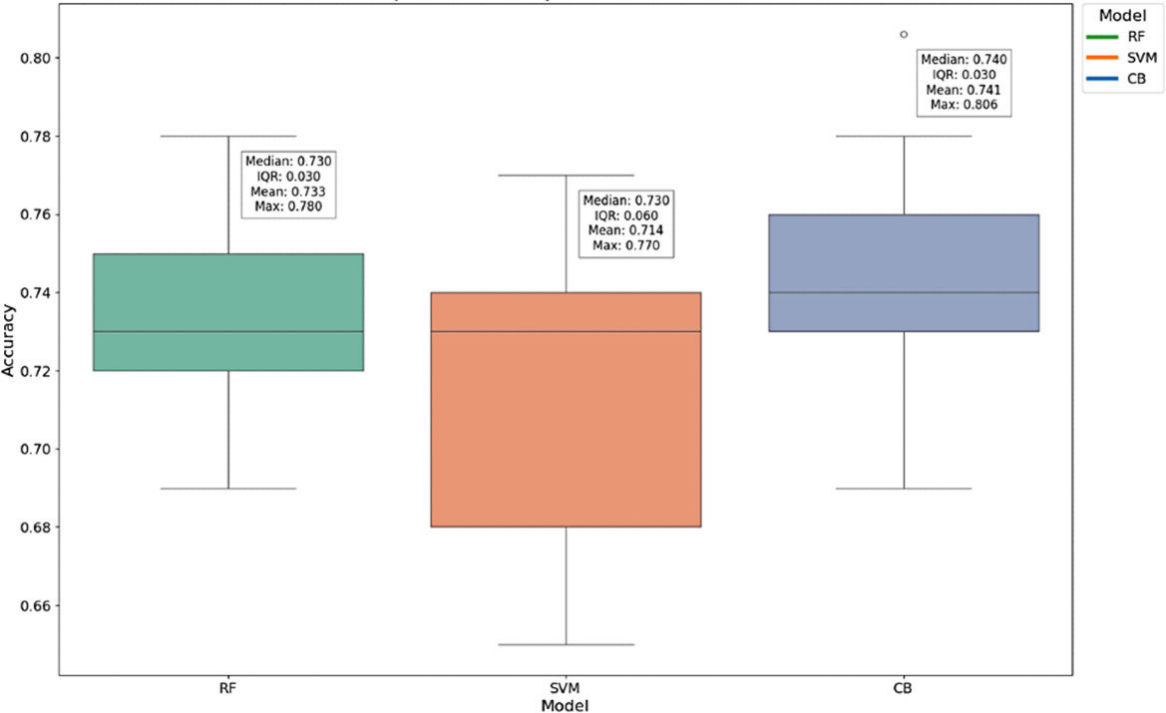
Box plot-based accuracy comparison of each machine learning model. CB: CatBoost; RF: random forests; SVM: support vector machine.

The dataset, comprising 693 observations, each containing 16 features in its feature vector, underwent experimentation using 3 different ML models to discriminate between MMSE ≤26 and MMSE 30 score groups. Figure 1 displays the accuracy of each model for all nCV combinations. The box plot analysis reveals that the CB model outperforms the RF and SVM models in accuracy. Specifically, the CB model achieves the highest median accuracy of 0.760, the highest mean accuracy of 0.752, and the highest maximum accuracy of 0.810. Additionally, the CB model shows the smallest variability in accuracy scores, as evidenced by the lowest IQR of 0.020. The RF model demonstrates a solid performance with a median and mean accuracy of 0.740, but it does not reach the same maximum accuracy as the CB model, peaking at 0.780. The RF model also has a moderate IQR of 0.030, indicating some variability in its performance. While still competitive, the SVM model shows the lowest median accuracy of 0.730 and the lowest mean accuracy of 0.721 among the 3 models. It also has a maximum accuracy of 0.760, which is lower than both RF and CB. The IQR of 0.030 is comparable to that of RF, indicating a similar level of performance variability.

The performance of the 3 ML models (CB, RF, and SVM) was evaluated using accuracy, precision, recall, and *F*_1_-score on both the training and test datasets. Table 2 summarizes these results. On the training set, RF achieved the highest scores across all metrics, followed closely by CB. However, on the test set, CB demonstrated the most balanced and generalizable performance, with an accuracy of 80.6%, precision of 77.4%, recall of 78.7%, and an *F*_1_-score of 78.0%. In contrast, RF showed signs of overfitting, indicated by a decline in test precision and accuracy despite maintaining high recall. SVM yielded moderate but consistent results across both datasets. These results suggest that CB is better suited for generalization in this context, likely due to its regularization features. RF, while highly effective on the training set, may have captured dataset-specific patterns that did not generalize well. SVM demonstrated stable but lower overall performance.

Table 3 shows the confusion matrix results associated with all models. The performance of 3 ML models, CB, RF, and SVM, was evaluated on both training and test datasets in terms of TP, FP, TN, and FN. Rather than focusing on raw counts, the interpretation emphasizes overall performance patterns. CB maintained a strong balance between correctly identifying both positive and negative cases. RF achieved high recall but also produced more FPs on the test set, which may limit its clinical precision. SVM showed more balanced errors, though with generally lower performance.

The hyperparameters and nCV combinations associated with each and the best-performing model are as follows:

- CB: 3X3nCV, (‘depth’: 4, ‘iterations’: 200, ‘l2_leaf_reg’: 5, ‘learning rate’: 0.1).
- RF: 4X7nCV, (‘max depth: 20, ‘min samples split’: 5, ‘n estimators’: 50).
- SVM: 8X5nCV, (‘C’: 10, ‘degree’: 3, ‘kernel’: 'poly’).

**Table 2.** The average performance metric scores for each machine learning model are displayed for the training and test sets.

Model	Precision (%)	Recall (%)	Accuracy (%)	*F*_1_-score (%)
Training set				
CB^a^	90.2	87.4	88.6	88.8
RF^b^	90.4^c^	92.6^c^	91.2^c^	91.5^c^
SVM^d^	75.8	69.1	72.7	72.3
Test set				
CB	77.4^c^	78.7	80.6^c^	78.0^c^
RF	68.5	82.0^c^	75.6	74.6
SVM	75.3	68.4	72.2	71.7

a CB: CatBoost.

b RF: random forests.

c Top-performing values.

d SVM: support vector machine.

**Table 3.** The confusion matrix outcomes for every machine learning classifier on both the validation and test sets.

	CB^a^		RF^b^ Predictedd)		SVM^c^	
	+	–	+	–	+	–
Training set						
Actual						
Positive (+)	249	36	264	21	195	90
Negative (–)	27	242	28	241	64	205
Test set						
Actual						
Positive (+)	48	13	50	11	46	15
Negative (–)	14	64	23	55	17	61

a CB: CatBoost.

b RF: random forests.

c SVM: support vector machine.

### Feature Importance

**Figure 2.**
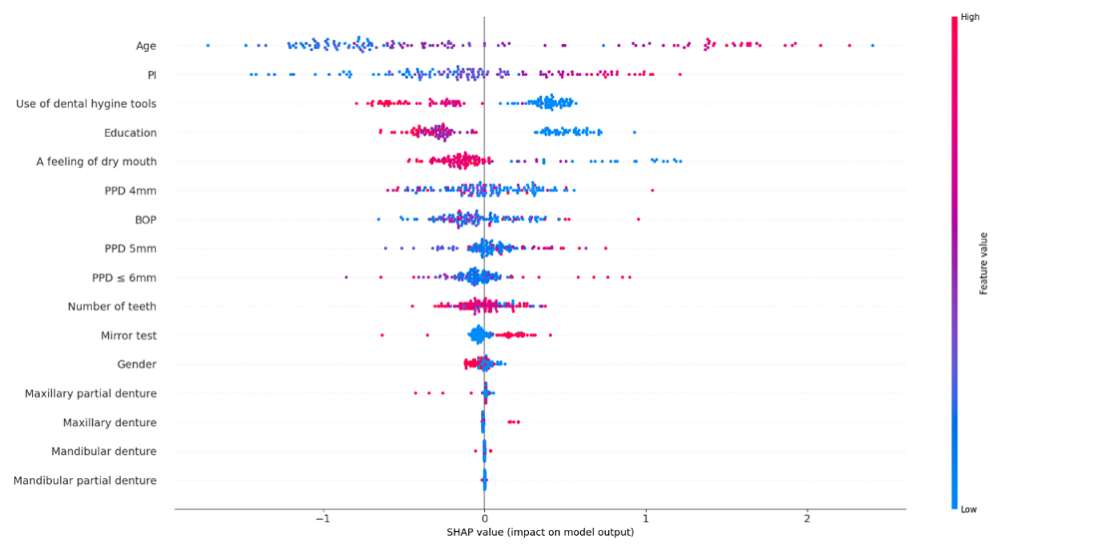
SHAP summary plot for model features. The plot illustrates the impact of each feature on the model’s output. BOP: bleeding on probing; PI: Plaque Index; PPD: probing pocket depth; SHAP: Shapley Additive Explanation.

The SHAP summary plot in Figure 2 illustrates the impact of various features on the model’s output. Each dot represents a Shapley value for a feature for an individual instance, and the color denotes the feature value, ranging from low (blue) to high (red). The SHAP summary plot reveals that age, PI, dental hygiene devices, education, a feeling of dry mouth, and PPD are critical in influencing the model’s predictions. The balance of these features, along with others like BOP and the number of teeth, determines the overall outcome predicted by the model. These findings indicate that age, PI, and PPD are significant predictors, with older age and higher PI correlating with an increased likelihood of having an MMSE score ≤26. Education appears to inversely affect the prediction, suggesting that higher education levels may be associated with better oral health practices. The number of teeth shows an inverse relationship, where having more teeth is linked to a lower risk of MMSE ≤26. Additionally, the presence of dental prosthetics and feelings of dry mouth positively influence the prediction, indicating a higher likelihood of MMSE ≤26. Gender shows a minimal impact, with the female gender slightly reducing the predicted MMSE ≤26 risk.

## Discussion

### Principal Findings

This study explored the potential of using ML technologies using oral health and demographic examination data to predict the probability of having MMSE scores of 30 or ≤26 in Swedish individuals older than 60 years. The main findings of the study were that all CB, RF, and SVM classifiers achieved high classification accuracies, but the CB classifier outperformed both RF and SVM classifiers with an average accuracy of 80.6%. Acceptable accuracy levels vary across studies [33-35], but it can be argued that all CB, RF, and SVM classifiers demonstrate strong performance, each exceeding 72% accuracy. This indicates that with specific nCV combinations and hyperparameter tuning, all classifiers can indicate whether the individual belongs to ≤26 MMSE scores or higher MMSE score (score=30) groups with varying grades of accuracy [36]. These findings hold the potential to identify individuals in need of support and treatment, benefiting not only them but also health care professionals and systems by optimizing treatment with fewer resources.

The performance evaluation based on the training and test set results is a common indicator of finding the unexpected accuracy relation of ML models [37]. The evaluation of the models reveals distinct differences in performance and generalization capabilities. On the training set, RF showed a well-balanced performance with strong metrics across the board, reflected in its Precision, Recall, Accuracy, and *F*_1_-score and its TP, TN, FP, and FN counts. The classifier CB performs better in the test set regarding the average performance metric, and SVM lagged significantly behind in both training and test sets. However, the general performance evaluation might differ from a clinical point of view when the higher recall is considered a more important parameter. In that case, RF with the highest TP values becomes the best classifier. FP could lead to unnecessary clinical evaluations or interventions, while FN could result in missed diagnoses, delaying treatment for cognitive impairment. When the goal of ML models is early detection and ensuring no cases of cognitive impairment are missed, minimizing FN is essential [38]. However, if the focus of these models is on reducing unnecessary interventions and managing health care resources efficiently, minimizing FP should be prioritized [39]. From a generalizability perspective, SVM shows the lowest performance drop between training and test, and CB indicates a second strong generalizability with a low-performance drop compared with the training and test set results. On the other hand, RF showed a decline in performance between the training and test sets. This suggests that SVM and CB are more suitable than RF for handling new, unseen data. Hyperparameter tuning was conducted through nCV using model-specific grids. For RF, parameters included the number of estimators, tree depth, and minimum samples per split. Despite this rigorous tuning, RF exhibited overfitting, reflected by its high training accuracy and reduced performance on the test set. This behavior suggests that RF, with its tendency to create deep and complex trees, may have captured noise or sample-specific patterns. In contrast, CB’s built-in regularization mechanisms, including L2-leaf regularization, contributed to more stable generalization. To address overfitting in future implementations, simpler models or additional regularization techniques such as pruning, reduced tree depth, or feature selection could be explored.

Oral health and demographic factors vary across different regions globally, and this method’s applicability is specific to Swedish conditions, which poses a limitation. However, a study from Taiwan [24], using oral health biomarkers and CB for cognitive decline classification predictions, yielded results akin to those presented in this study. In their model [24], crucial features included daily social interaction, oral diadochokinesis, PI, saliva protein, and age. In contrast, this study benefits from a more extensive and balanced dataset, presenting absolute average values rather than weighted results. Additionally, to mitigate overfitting risks, nCV was used to train ML classifiers across various k-fold combinations to mitigate overfitting risks in ML classifiers across various k-fold combinations. Furthermore, these studies collectively highlight oral health and demographic factors that vary across different regions globally, and this method’s applicability is specific to Swedish conditions. Additionally, while the use of 10 × 10 nCV ensures an unbiased estimate of generalization performance within the dataset, the importance of validating models on external data is well recognized. Internal validation alone cannot capture the variability introduced by different clinical environments, geographic regions, or data acquisition protocols. External validation on independent cohorts or through integration into routine dental examinations would be required to evaluate robustness. Such efforts would help assess the model’s applicability under real-world variability and determine its readiness for broader clinical use.

The implications drawn from the SHAP summary plot underscore critical insights into feature relevance concerning cognitive health assessments via ML models. The clear demarcation between the groups of MMSE scores ≤26 and 30 across influential variables like age suggests that such demographic factors hold substantial weight in predictive analytics. Notably, age emerges as a pivotal factor, with higher age correlating strongly with lower cognitive health scores. This correlation is also highlighted in other studies that consistently demonstrated that MMSE scores tend to be lower for older individuals and decline over time [40]. The SHAP summary plot highlights age, PI, and use of dental hygiene tools as the most influential predictors of MMSE classification. Older age and higher plaque levels were associated with increased likelihood of MMSE ≤26, suggesting that both biological aging and reduced oral hygiene maintenance contribute to cognitive risk [20,41]. In particular, individuals with lower use of interdental hygiene tools exhibited stronger SHAP effects, indicating a potential interaction between age-related decline in motor or cognitive function and oral hygiene behaviors. This aligns with findings that individuals with cognitive impairment often experience challenges in maintaining oral hygiene due to reduced executive functioning and manual dexterity [7,42]. Features such as dry mouth and education also showed moderate predictive strength, consistent with their known associations with polypharmacy and cognitive reserve, respectively [41]. In contrast, features like BOP, gender, and presence of dentures showed minimal SHAP influence, suggesting weaker or less consistent relationships within this sample. These findings suggest that age-related decline may act synergistically with deteriorating oral hygiene habits, offering further evidence for behaviorally anchored early risk screening in clinical practice.

Given that MCI resides on the border between healthy aging and neurocognitive illness, individuals with MCI may fluctuate between MMSE scores ≤26 and 30 [43]. About 50% of individuals with MCI receive a neurocognitive diagnosis within 3 years, with a 2-step decrease in MMSE score within 12 months indicating disease onset. Recommended thresholds for the MMSE can be expressed as 0‐22, indicating dementia; 23‐26, MCI [44]; and 27‐30, indicating normal cognitive function [7,42]. In this study, the threshold MMSE≤26 was used. This criterion was adopted in the study, resulting in the exclusion of MMSE scores of 27, 28, and 29 from calculations. This limit has been chosen as 2 different studies have found that changes of 2 to 3 points indicate reliable changes in the MMSE test value at the 90% confidence level [28,45]. However, it is important to note that this regulatory threshold may limit the study, potentially leading to missed indications. The exclusion of MMSE scores of 27, 28, and 29 may also affect generalizability. One of the strengths of this study is the use of ML as the methodology. Recent studies in ML have shown significant promise in predicting various disorders, such as dementia [46,47], chronic obstructive pulmonary disease [48], cancer [49], and Parkinson disease [50]. The used tree-based models, CB and RF, and the distance-based model SVM allow comparability in the case of possible effects of outliers in the dataset, where the tree-based models are known to be more resistant to imbalance in the dataset than the distance-based models [51-53]. Despite this study seeking a balanced data set, there is a heterogeneity regarding ages represented in the dataset where some ages might be overrepresented, which can be seen as a limitation in this study. Another limitation is the low number of participants and the recruitment that took place in a small county in southern Sweden, which is an obstacle to making conclusions from a broader perspective that represents more variety in demographic and regional differences. However, in relation to previous research, this study includes a larger number of participants, thereby increasing the generalizability of the findings. Another notable difference concerns the threshold used to distinguish between the MMSE ≤26 and MMSE 30 groups; in this study, a higher threshold was applied. Since the dataset of 693 observations is relatively small and may not be representative of a larger population, it may affect generalizability. In addition, even if the groups are relatively balanced, small differences in group size (339/693, 49% vs 354/693, 51%) can affect model performance and bias. These limitations can be overcome by expanding the data set by including divergent and wider participants’ data from larger geographical areas in upcoming studies. Future research may also address the performance of capturing changes over time for monitoring purposes, highlighting the need for longitudinal studies. Although the findings demonstrate strong internal performance, several practical challenges constrain the model’s readiness for clinical use. These include variability in oral health assessment protocols, limited interpretability of model outputs for nontechnical users, and the absence of integration pathways with existing clinical information systems. SHAP plots provide valuable transparency but may need adaptation for clinician-facing use. Implementation would require standardization of input data, compatibility with electronic health records, and interdisciplinary development efforts. Further investigation is needed to evaluate clinical usability and workflow alignment.

Other limitations of the study are that only baseline data were used from longitudinal studies, meaning that potential changes over time may be missed. Only 16 parameters were selected, which means that other important parameters that could affect the results may have been omitted. The oral health features used in the models were selected based on their consistent availability across both datasets and their previously reported clinical relevance to cognitive function. These features, such as PI, number of teeth, and dry mouth, may reflect both localized oral conditions and systemic factors, including frailty, polypharmacy, and reduced self-care capacity. Such conditions have been associated with cognitive impairment in older adults. Other potentially relevant features, like microbial profiles or periodontal attachment levels, were not included due to data limitations, but future work could incorporate these variables in extended datasets.

### Conclusion

This study suggests that oral health parameters, demographic data, and ML techniques offer a potential tool for screening MMSE scores for individuals aged 60 years and older. The data used as inputs for ML classifiers contains sufficient information to differentiate between MMSE scores ≤26 and 30.

It is important to include both demographic and oral health-related factors in predictive models. Despite some limitations, the study offers valuable insights for future research and potential clinical applications. To ensure the robustness and generalizability of the model, further external validation and adaptation to different clinical settings are required.

## Abbreviations

AD: Alzheimer Disease
BOP: bleeding on probing
CB: CatBoost
FN: false negative
FP: false positive
MCI: mild cognitive impairment
ML: machine learning
MMSE: Mini-Mental State Examination
nCV: nested cross-validation
PI: Plaque Index
PPD: probing pocket depth
RF: random forest
SHAP: Shapley additive explanation
SMART4MD: Support Monitoring and Reminder Technology for Mild Dementia
SNAC-B: Swedish National Study on Aging and Care
SVM: support vector machine
TN: true negative
TP: true positive
